# Neuroendocrinal, Neurodevelopmental, and Embryotoxic Effects of Recombinant Tissue Plasminogen Activator Treatment for Pregnant Women with Acute Ischemic Stroke

**DOI:** 10.3389/fnins.2016.00051

**Published:** 2016-02-25

**Authors:** Anna Steinberg, Tiago P. Moreira

**Affiliations:** ^1^Department of Neurology, Karolinska University HospitalStockholm, Sweden; ^2^Stroke Research Unit, Department of Clinical Neuroscience, Karolinska InstitutetStockholm, Sweden

**Keywords:** rTPA, alteplase, brain development, toxicity, haemorrhagic, intravenous, teratogenic, uterine

## Abstract

Thrombolysis with recombinant tissue plasminogen activator (rTPA) was the first evidence-based treatment approved for acute stroke. Ischemic stroke is relatively uncommon in fertile women but treatment is often delayed or not given. In randomized trials, pregnancy has been an exclusion criterion for thrombolysis. Physiologic TPA has been shown to have neuroendocrine effects namely in vasopressin secretion. Important TPA effects in brain function and development include neurite outgrowth, migration of cerebellar granular neurons and promotion of long-term potentiation, among others. Until now, no neuroendocrine side-effects have been reported in pregnant women treated with rTPA. The effects of rTPA exposure in the fetus following intravenous thrombolysis in pregnant women are still poorly understood. This depends on low case frequency, short-duration of exposure and the fact that rTPA molecule is too large to pass the placenta. rTPA has a short half-life of 4–5 min, with only 10% of its concentration remaining in circulation after 20 min, which may explain its safety at therapeutically doses. Ischemic stroke during pregnancy occurs most often in the third trimester. Complication rates of rTPA in pregnant women treated for thromboembolic conditions and ischemic stroke were found to be similar when compared to non-pregnant women (7–9% mortality). In embryos of animal models so far, no indications of a teratogenic or mutagenic potential were found. Pregnancy is still considered a relative contraindication when treating acute ischemic stroke with rTPA, however, treatment risk must be balanced against the potential of maternal disability and/or death.

## Neuroendocrine effects of tissue plasminogen activator

Tissue plasminogen activator (TPA) is a serine protease that converts plasminogen into the fibrinolytic enzyme plasmin thus promoting fibrin dissolution in blood clots (Carmeliet et al., 1994). Endothelial cells are the major source of circulating TPA, which is released upon stimulation by factor X-a, bradykinin, fibrin, platelet activating factor, and thrombin (Booyse et al., 1986; Emeis, 1992). Other triggers of TPA released into the bloodstream include among others, anxiety, exercise, surgery, and electroconvulsive therapy, however, these conditions are also coupled to catecholamine release, which may thus be the shared trigger mechanism for both direct TPA release and TPA release from endothelial cells in these conditions. In support of this notion, TPA was demonstrated to be co-expressed and trafficked simultaneously with noradrenaline in the chromaffin cells of the adrenal glands (Parmer et al., 1993). Chromogranin A is one soluble protein that is co-released with TPA and catecholamines. It works as prohormone which, when cleaved into active peptides, inhibits the further release of catecholamines (Parmer et al., 1993). Experiments with knockout mice lacking TPA provided further evidence for a role of TPA in behavioral stress responses and catecholamine release. These mice exhibit deficient stress-induced anxiety behavior (Pawlak et al., 2003, 2005) and show anxiety-like behavior after intracerebroventricular injection of corticotrophin releasing factor (Matys et al., 2004).

TPA-cleaved plasmin regulates proteolysis of among other, laminin, collagen IV, proteoglycans, pro-brain-derived neurotrophic factor (pro-BDNF), and protease activated receptor-1 (Dityatev and Schachner, 2003; Pang et al., 2004). TPA has been shown to directly interact with low-density lipoprotein receptor-related protein (LRP) leading to phosphorylation of mitogen-activated protein kinase (MAPK) 1 and extracellular signal-regulated kinases-1 and 2 (Zhuo et al., 2000; Hu et al., 2006). In turn, serpins (including the CNS variant neuroserpin) and plasminogen activator inhibitor PAI-1 and -2 are the main inhibitors of the serine protease family including TPA, urokinase-type plasminogen activator (uPA), plasmin, and thrombin (Yepes and Lawrence, 2004).

Endogenous TPA is widely distributed in the neuroendocrine system. In the neuroendocrine cells of the hypophysis, the magnocellular neurons of the hypothalamic supra-optic nucleus, the chromaffin cells of the adrenal medulla, thyroid and parathyroid glands, endogenous TPA first enters the endoplasmic reticulum where it binds to a signal peptide and is transported through the Golgi complex. It is then released either via the regulated secretory pathway (vesicular) or the constitutive secretory pathway (direct release) (Kelly, 1985). Although there is one regulated secretory pathway, TPA gets rapidly released from storage vesicles originated from the Golgi complex and appears to be mediated by calcium ion influx (Gualandris et al., 1996). A similar mechanism of TPA co-release with parathyroid hormone has been described in parathyroid cells (Bansal and MacGregor, 1992).

In the brain, wide expression of neuronal TPA and the plasmin inhibitor neuroserpin are found in the developing and adult nervous system and have been shown to play a role in neuronal plasticity (Lee et al., 2015). TPA expression predominate in the lobar hemispheres, thalamus, medulla oblongata, and mesencephalon whereas neuroserpin, although also overlapping with TPA in the lobar hemispheres and mesencephalon, is more abundant in the spinal cord, substantia nigra and Purkinje cells (Teesalu et al., 2004). Moreover, TPA mRNA expression is seen in ventricular ependymal cells and meningeal blood vessel cells (Hashimoto et al., 1998).

In particular, a role of TPA in the neurohypophysis has been proposed for the osmotic regulation of body fluids. The antidiuretic hormone vasopressin (arginine-vasopressin or AVP) is synthesized in the magnocellular neurons of the hypothalamic supra-optic nucleus and paraventricular nucleus, and packed into neurosecretory granules, which are transported through their axons over to the neurohypophysary terminals (Miyata and Hatton, 2002). TPA immunoreactivity was observed at neurosecretory granules of vasopressin-positive magnocellular terminals and that of plasminogen was seen at astrocytes. With electron microscopy, Imamura et al. were able to show a specific localization of TPA at neurosecretory granules containing vasopressin, indicating that TPA is co-released along with the exocytosis of vasopressin and might be an early regulator of vasopressin release (Imamura et al., 2010).

TPA has been implicated in neurite outgrowth of neuronal cultures (Pittman et al., 1989), neuronal regeneration, migration of cerebellar granule neurons (Seeds et al., 1995), and prohormone synthesis (Sappino et al., 1993). TPA is capable of potentiating N-methyl-D-aspartate (NMDA) receptor activation by cleaving the NMDA receptor 1 (NMDAR1) subunit (Nicole et al., 2001). The significance TPA-induced cleavage of NMDAR1, as well as cleavage of pro-BDNF by plasmin is particularly relevant for learning and memory. On the one hand, TPA knockout (KO) mice show reduced maintenance of the long-term potential in the hippocampal CA1 area and exhibit less open-field exploration and poor performance in a context-conditioning task (Calabresi et al., 2000). On the other hand, mice overexpressing TPA show an enhancement of the long-term potential in the hippocampus with improved performance in spatial navigation learning tasks (Baranes et al., 1998; Madani et al., 1999). Long-term depression is absent in the striatum of TPA KO mice and has been coupled with decreased rearing activity and object exploration, as well as with poorer performance in a two-way active avoidance task (Calabresi et al., 2000). In 3 month-old Fischer rats, increased TPA mRNA expression is detected in Purkinje cerebellar neurons following 1 h of complex motor task learning in rats (Seeds et al., 1995). Ocular dominance plasticity in the visual cortex was also shown to be related to TPA and plasmin activities (Müller and Griesinger, 1998; Mataga et al., 2004). The authors have also suggested that a cascade of plasmin generated by TPA may selectively mediate cortical plasticity, perhaps via structural remodeling of axons (Müller and Griesinger, 1998; Mataga et al., 2004). Evidence for a function of TPA and the brain-specific protease inhibitor neuroserpin in regulating axonal growth has come from studies of cultured cells (for a recent review see Lee et al., 2015). Hashimoto and colleagues found evidence supporting TPA involvement in long-lasting cortical plasticity following psychotomimetic administration in the rat by observing increased mRNA expression in prefrontal cortex neurons projecting to the medial striatum (Hashimoto et al., 1998).

Finally, ischemic damage is suggested to lead to excess endogenous TPA activity in the brain and contribute to neurodegeneration via extracellular matrix degradation, microglia activation, and blood brain barrier leakage (Lee et al., 2015). Neuroserpin-knockout mice have worse ischemic damage and neurological outcomes than controls, with the effects attributed to TPA-mediated activation of microglia (Gelderblom et al., 2013). Experimental intravenous (exogenous) TPA administration was shown to increase cerebrovascular permeability and decrease cerebrovascular resistance (Tsirka et al., 1995; Yepes et al., 2003; Nassar et al., 2004).

## Treatment with recombinant TPA in pregnant women

About 85% of all strokes are ischemic and the remaining are hemorrhagic. Spontaneous reperfusion may occur through endogenous release of plasminogen activator, which stimulates plasmin formation from plasminogen. For larger occlusions this release seems insufficient to induce reperfusion in time to avoid a cerebral lesion. Administration of alteplase, a recombinant tissue plasminogen activator (rTPA) as an injectable drug, which is commonly used to treat myocardial infarction, stroke and thrombosis, is thus one method to enhance this endogenous procedure (for a recent review see Prabhakaran et al., 2015). Acute ischemic stroke in pregnant women occurs most commonly in the third trimester and is potentiated by an increased pro-coagulant state during pregnancy, higher risk for cervical and intracranial artery dissection peri-partum, as well as by persistent foramen ovale and other underlying cardiac conditions.

The thrombolytic effect of rTPA varies among species. Humans are proposed to have a more sensitive fibrinolytic system to the effects of rTPA (Korninger and Collen, 1981). Thus, in humans the effective and safe dose for acute stroke treatment is 0.9 mg/kg. In rats, a dose of 1.8 mg/kg up to 10 mg/kg induced recanalization of carotid artery occlusion in 17–71%, whereas in humans this is only achieved in 10–30% of cases. The 1.8 mg/kg dose in the rat is proposed to be equivalent to the human dose of 0.9 mg/kg in terms of efficacy (Tomkins et al., 2015). In rabbits, a dose of 5 mg/kg—but not of 3 or 10 mg/kg—is capable of dissolving an intracerebral clot embolized from the carotid artery (Bednar et al., 1993). Until now, randomized controlled trials have excluded pregnant women and patients with increased hemorrhage risk from participation in studies regarding thrombolysis treatment. In animals rTPA does not cross the placenta and there has been no evidence of teratogenicity (Chan et al., 2000; Leonhardt et al., 2006; De Keyser et al., 2007). To date there are no reports on rTPA being able to cross the human placenta. In 2006, Leonhardt et al. had reviewed 18 cases of pregnant women treated with rTPA for other thromboembolic conditions, mainly pulmonary embolism, deep vein thrombosis and thrombosed cardiac valve prosthesis and 10 cases of pregnant women treated with rTPA for acute stroke, including an own stroke case (Leonhardt et al., 2006). Good maternal neurological outcome was reported for all but two mothers who died (one with stroke, the other with mitral valve thrombosis) and one who developed cerebral infarction. Ineffective thrombolysis or partial arterial recanalization was reported in four mothers. Twenty children were born with good outcome, however, there were two spontaneous abortions, three pregnancy interruptions owing to maternal cause and one infant died at 2 weeks' post-partum. Thus, there was a similar rate of complications in pregnant women compared to non-pregnant women, with mortality at about 7% for the mother and about 23% for the child (half of the child losses occurred in three stroke cases; the other half in two pulmonary embolisms and one valve thrombosis). Possible explanations for child loss not addressed by this review may include the severity of the underlying maternal medical condition rather than a direct effect of rTPA treatment alone. Interruptions of pregnancy may also have been carried following medical decision. Later in 2006, Wiese et al. reported use of intravenous rTPA thrombolysis in a pregnant woman with acute cardioembolic stroke. The patient improved clinically, did not develop complications after receiving rTPA, and at 37 weeks' gestation, delivered a healthy infant (Wiese et al., 2006). Yamaguchi et al. reported a 36 year-old woman, who was 18 weeks pregnant and developed a sudden onset of motor aphasia and hemiparesis on the right side. The NIH stroke scale was 6, and the brain MRI indicated occlusion of the left middle cerebral artery branches. She was treated with intravenous rTPA with subsequent recanalization of the occluded left middle cerebral artery branches. The symptoms disappeared within a few hours after treatment. She delivered a healthy infant without any apparent complications (Yamaguchi et al., 2010). There are further cases of successful use of rTPA in pregnant women with acute stroke, the majority in the third trimester of pregnancy (Dapprich and Boessenecker, 2002; Elford et al., 2002; Johnson et al., 2005; Murugappan et al., 2006). In 2012, Li et al. reported one own stroke case and reviewed 10 previously published stroke cases. They reported good to complete recovery in 10 mothers and one death during endovascular treatment, resulting in the delivery of eight healthy infants, two medical terminations of pregnancy, and one fetus death (Li et al., 2012). In 2013 and 2014, two additional stroke cases with good outcome for the mothers and the fetuses were reported by Tassi and Ritter, respectively (Tassi et al., 2013; Mantoan Ritter et al., 2014). The most recent case of successful rTPA treatment in a pregnant woman at 39 weeks of gestation with normal delivery was reported in 2015 (Ritchie et al., 2015). So far, only one mother treated with rTPA for acute stroke suffered a significant uterine bleeding complication (Demchuk, 2013), however, caution about bias publication should be taken into account when reviewing case reports. Intravenously administered rTPA has a high affinity for fibrin strands and a short half-life of 4–5 min via liver metabolism, with only 10% of its concentration remaining in circulation after 20 min, which may explain its safety at therapeutic doses.

In menstruating women, Wein et al. described five subjects in the active arm of the National Institute of Neurological Disorders and Stroke (NINDS) intravenous thrombolysis trial, who were coded as actively menstruating. One subject who had a 1-year history of dysfunctional uterine bleeding required urgent uterine artery ligation. The authors also reported a case of a woman requiring transfusion after intravenous thrombolysis for acute ischaemic stroke (Wein et al., 2002).

So far, no neuroendocrine side-effects have been reported in pregnant women treated with alteplase.

## Effects of exposure to rTPA in brain development

As stated above, intravenous rTPA is too large a molecule (7200 KDa) to be able to pass the placental blood barrier. From clinical reports of IVT-treated pregnant women with stroke, there have been no signs of brain development issues on the surviving fetuses. The European Medicines Agency license for alteplase includes information on embryotoxicity (in the form of embryolethality and growth retardation) in pregnant rabbits given 3 mg/kg alteplase, which is over 3 times the therapeutical dose (0.9 mg/kg). However, no teratogenic effects were observed in animals treated with i.v. therapeutical doses and no effects on peri- or post-natal development or fertility were observed in rats treated with doses up to 10 mg/kg (Kojima et al., 1988) In subchronic toxicity studies in rats and marmosets no unexpected adverse effects were observed. No indicative signs of mutagenesis were found (preclinical safety data included in the European license documentation) (EMEA, 2002).

## Ongoing studies

Uncertainty whether fertile women with potential or known pregnancy should be treated may delay or halt thrombolysis and worsen stroke outcome. Maternal hemorrhagic complications have been reported in 8% with systemic thrombolysis across the spectrum of clinical thromboembolic indications (Cronin et al., 2008). More specifically, mortality in 172 pregnant women treated with a potent thrombolytic agent, streptokinase, was reported at 1.2% (Turrentine et al., 1995), which is far lower than the 9.5% mortality owed to stroke alone in pregnant women (Ritchie et al., 2015). Thus, considering this limited risk, pregnancy should not be considered an absolute contraindication. The risk during pregnancy must be balanced against the potential of a disabled outcome without treatment (Demchuk, 2013). To explore the safety of thrombolysis in pregnant women with acute stroke, and indeed within the whole group of fertile women, the Safe Implementation of Treatments in Stroke International Stroke Thrombolysis Register (SITS-ISTR), a prospective, international, observational registry for medical centers documenting stroke treatments (Wahlgren et al., 2007) has been expanded to include specific questions for women in the age group 13–50. The aim is to systematically collect data, to contribute to knowledge about treatment safety for these women, and to explore whether treatment in pregnant women, or indeed all women in fertile age is safe and not delayed. We estimate that a number of women will be treated despite pregnancy, partly because the condition was not considered when treatment was initiated, or because the potential benefit was judged higher than the risk. The overall aim of the study, Safe Implementation of Treatments in Stroke-Fertile Women Stroke Thrombolysis Study (SITS-FW), is to determine if pregnancy and even menstruation constitutes any safety issue when treated with thrombolysis, or if these patients can be given the same opportunity for treatment as other patients.

## Conclusions

Pregnancy is still considered a relative contraindication for intravenous thrombolysis with rTPA for acute ischemic stroke within 4.5 h of symptom onset. However, the present and previous reviews indicate a similar maternal safety profile compared with non-pregnant women. This should be further analyzed in future prospective studies. It is reasonable to weigh in the benefit of rTPA vs. the risk for the fetus in this patient group and offer treatment for moderate to severe disabling stroke, particularly if there is no access to endovascular treatment. With the current ongoing implementation of mechanical thrombectomy for acute ischemic stroke in routine practice (Wahlgren et al., 2016), we expect more pregnant women to benefit from acute reperfusion strategies that may or not include intravenous thrombolysis in addition to mechanical thrombectomy for large vessel occlusions.

## Author contributions

AS, Planned litterature review, wrote first draft with references. TM, Planned litterature review, wrote abstract, edited first draft, and wrote final version.

### Conflict of interest statement

AS has no disclosures. TM has received travel and lecture grants from Boehringer-Ingelheim.
